# Notes and News

**Published:** 1896-06-20

**Authors:** 


### Notes and News.

(Collected and Communicated.)

M. Laurent-Marie-Chalery has bequeathed 5,000 francs to the Pasteur Institute in Paris.

Dr. Thomas Barlow, F.R.C.P., &c., physician to the University College Hospital and to the Hospital for Children, Great Ormond Street, has been appointed Physician in Ordinary to the Queen's Household in place of the late Sir John Russell Reynolds.

It is proposed to erect a new statue to Jenner in some prominent place in the metropolis, that already existing in Kensington Gardens not being considered of sufficient importance to do honour to so distinguished a man.

Lord Amherst of Hackney has given £100 towards the completion of the purse of £1,000, which was started, with a similar donation, by the Duke of Westminster in aid of the North-Eastern Hospital for Children, and is to be presented to the Duchess of Connaught at the Rose Fête at Queen's Hall on the 23rd inst.

We are pleased to note from the latest balance-sheet of the Surgical Aid Society that its administration expenses, *i.e.*, salaries and office expenses, amount to only 21 per cent. of the expenditure. It is usual to find that considerably more than one-half of the expenditure of societies formed to supply surgical appliances to the poor is due to these items.

Hospital Sunday in London was a particularly brilliant day, and the churches were thronged with specially large congregations. The pleading from the pulpit was in most cases exceptionally eloquent, and it is to be hoped that the full result of the collection will even exceed the expectations of those who have the interests of the hospitals at heart. The morning service at St. Paul's Cathedral was attended by the Lord Mayor and the Sheriffs in state, who also attended the afternoon service in Westminster Abbey.

Whilst noting that the cholera epidemic, both in Alexandria and Cairo, is steadily diminishing, we regret to say that the plague at Hong Kong seems to be slightly gaining ground.

We have received a copy of the festival number of "Guy's Hospital Gazette." It is beautifully got up, and contains, amongst interesting illustrations and letterpress, a beautiful etching of Guy's Hospital at the present time.

The Emperor of Austria has always shown himself exceedingly ready to do honour to medical science. Quite recently Professors Leussen and Wiederhofer have been singled out for distinction. Professor Leussen, who is a high authority on internal medicine, has been created a Privy Councillor, and Professor Wiederhofer has received a special letter of thanks from the Emperor for his attendance on the deceased Archduke Karl Ludwig.

A cottage hospital has just been opened at Tavistock. The institution is the gift of the late Miss A. Herring, who left a legacy of about £4,000 for this purpose. The Duke of Bedford, co-operating with this generous action, has provided a site free of cost. The building is of two storeys, the wards facing south. The men's ward is situated on the ground floor, which also contains the operating-room, the matron's sitting-room, dispensary, and the administration offices. The women's ward and three smaller ones for private cases, together with the sleeping accommodation for matron and servants, occupy the upper floor.

The British Ophthalmic Hospital at Jerusalem, which belongs to the Grand Priory of the Order of the Hospital of St. John of Jerusalem in England, during the nine months ended September 29th, 1895, treated 355 in and 3,486 out-patients. These patients came from all parts of the country, from Gaza in the south to Nazareth in the north, from the sea-coast to the Jordan valley, and from the Bedouin tribes and other inhabitants east of the Jordan and south of Palestine. The seventeen beds in the hospital are full for the greater portion of the year, and usually the hospital has to put up nineteen patients. The committee make a special appeal to the members of the Order, stating that if every one of them would subscribe an average of one guinea yearly the work of the hospital could be carried on without loss.

The nominations which have been received for the forthcoming election to the Council of the Royal College of Surgeons are as follows: Mr. Thomas Bryant, Mr. Pickering Pick, Mr. George Pollock, Mr. Davies Colley, Sir William Dalby, Mr. Clement Lucas, Mr. Edmund Owen, and Mr. Walsham. There are but three vacancies, and it is a matter of no small importance that the choice of the Fellows should fall on the right men. Among others it seems very desirable that Mr. Clement Lucas should obtain a seat on the Council. He is understood to be in favour of all the constituents being represented on the Council by members of their own bodies; he is also in favour of various other much-needed reforms, and is well known as a surgeon to one of the best metropolitan schools. It is greatly to be hoped that the many Fellows of the College of Surgeons who were educated at Guy's Hospital will make use of their voting papers to secure the return of Mr. Clement Lucas as a representative of their old school.

